# Reflection of medical error highlighted on media in Turkey: A retrospective study

**DOI:** 10.12669/pjms.325.10042

**Published:** 2016

**Authors:** Oguz Isik, Gamze Bayin, Ozgur Ugurluoglu

**Affiliations:** 1 Oguz Isik, PhD. Department of Health Administration, Faculty of Economics and Administrative Sciences, Hacettepe University, Beytepe Campus, Ankara, Turkey; 2 Gamze Bayin, Msc. Department of Health Administration, Faculty of Economics and Administrative Sciences, Hacettepe University, Beytepe Campus, Ankara, Turkey; 3 Ozgur Ugurluoglu, PhD. Department of Health Administration, Faculty of Economics and Administrative Sciences, Hacettepe University, Beytepe Campus, Ankara, Turkey

**Keywords:** Medical error, Media

## Abstract

**Objective:**

This study was performed with the aim of identifying how news on medical errors have be transmitted, and how the types, reasons, and conclusions of medical errors have been reflected to by the media in Turkey.

**Methods:**

A content analysis method was used in the study, and in this context, the data for the study was acquired by scanning five newspapers with the top editions on the national basis between the years 2012 and 2015 for the news about medical errors. Some specific selection criteria was used for the scanning of resulted news, and 116 news items acquired as a result of all the eliminations.

**Results:**

According to the results of the study; the vast majority of medical errors (40.5%) transmitted by the news resulted from the negligence of the medical staff. The medical errors were caused by physicians in the ratio of 74.1%, they most commonly occurred in state hospitals (31.9%). Another important result of the research was that medical errors resulted in either patient death to a large extent (51.7%), or permanent damage and disability to patients (25.0%).

**Conclusion:**

The news concerning medical errors provided information about the types, causes, and the results of these medical errors. It also reflected the media point of view on the issue. The examination of the content of the medical errors reported by the media were important which calls for appropriate interventions to avoid and minimize the occurrence of medical errors by improving the healthcare delivery system.

## INTRODUCTION

In the health system, the technologies and the complex combination of human interaction has resulted in significant benefits for patients, but it can also bring along inevitable risks causing undesired results.^1^ Medical errors that are a probable threat to patient safety involve undesired events of the healthcare system and constitute an important topic for medical services.^2^,^3^

Medical error may be defined as any problems in the process of medical services,^4^ or failure of a planned health care action or utilizing from an incorrect health care action plan to overcome a health problem.^5^ The Joint Commission^6^ defined a medical error as implementations resulting in death, or permanent or temporary damage in the vital functions of a patient. IOM (Institute of Medicine) expressed prevalently encountered medical errors, in a report issued in 1990, as the wrong drug effects, inappropriate transfusions, surgical injuries, wrong site surgeries, negligence oriented injuries and deaths, falls, burns, and wrong patient identifications during the presentation of the medical service.^7^

Many studies have revealed the prevalence of avoidable medical errors.^7^-^9^ The reduction in medical errors will create a significant impact on important concepts such as the improvement of patient safety and the reduction of health care cost also.^10^ The reduction, prevention, or noticeability of medical errors prior to causing damage are associated with the notification of errors, revealing their reasons, and strategy to avoid them.^11^ The media has great influence on patient safety improvement efforts.^12^ News published in newspapers constitutes a framework for the outlining of events and issues concerning society. Thus, it influences the perception of society for events and issues.^13^ Therefore, it has been considered that the media will shed light on medical errors’ implementations, which are multidimensional in terms of social and economic issues, and will provide an insight into both the nature and size of the medical errors.^14^,^15^

The media have the power to affect public opinion about many medical services- including medical errors- and to affect public policies on such issues.^16^,^17^ The findings in respect to the studies performed by Grilli et al.^18^ and Stebbing et al.^19^ revealed that the media could be used as an intervention in order to improve patient safety through changing the perceptions and behaviors of the patients and medical services providers. The study intended to reveal how the news about medical errors were discussed, and how they were transmitted to the readers via the media in Turkey. The identification of how the framework of medical errors was outlined by media is considered to be important in terms of issues such as creating awareness in the community, policy configuration, and the establishment of patient safety approaches also.

## METHODS

This research was performed through a content analysis method, a qualitative research approach, with the intention to identify how medical error news was transmitted by the media in Turkey. In this context, the data for the study was acquired by scanning the news about medical errors in five newspapers (Zaman, Hurriyet, Posta, Sozcu, and Sabah) with the top editions on the national basis between the years June 1, 2012-June 1, 2015.

The data was acquired from the websites of the aforementioned five newspapers. While scanning; the following key words created on the basis of definitions of medical errors were used: “medical error”, “doctor’s/physician’s error”, “nurse’s error”, “doctor’s/physician’s negligence”, “wrong site surgery”, “wrong operation”, “incorrect/wrong drug”, “falling off a stretcher”, “wrong diagnosis/recognition”, “test errors”, “overdose”, “the delaying of treatment “and “unnecessary/excessive treatment”.

**Fig.1:**
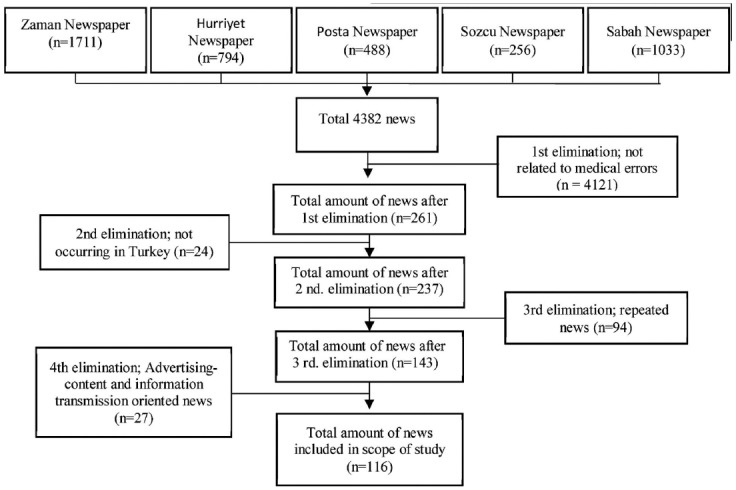
Flowchart in respect to the selection of the news to be discussed in the scope of the study.

For the scanning oriented resulted news, a specific selection criteria was used. The basic criteria for the news selection was that the news be directly related to the subject of medical error and that they were made within the borders of Turkey. In addition, advertising content and information transmission oriented news (expert opinions, meetings and panels, and information for implementation) and repeated news were not included within the scope of the research (Fig.1).

One hundred sixteen news reports published by the media were selected for this study after eliminating all those which did not fall within the scope of this research. Then we looked at the type of medical error, who was affected with the medical error and who was considered responsible for these medical errors, the place where the medical errors occurred, and what were the results.

## RESULTS

The types of medical errors that occurred within the scope of the research and findings in regard to the various parties are given in Table-I. Vast majority of medical errors (40.5%) resulted from the negligence of the medical staff, and the errors resulting from system inability constituted the least (1.7%) type of error that occurred While it was noticed that medical error was caused by physicians in the ratio of 74.1%; a large majority of people exposed to medical error were both female (66.1%) and people in the age range of 0 to 16 and 17 to 33 (70.8%) (Table-I).

**Table-I:** Types of medical errors that occurred and the findings relating to the various parties (n=116).

		*n*	*%*
The Type of Medical Error that Occurred	Negligence of Medical Staff*	47	40.5
	Wrong Drug Implementation	20	17.2
	Wrong Operation	15	12.9
	Wrong Diagnosis	14	12.1
	Wrong Intervention	13	11.2
	Infection	5	4.3
	System Inability Based Error	2	1.7
Those who Caused the Medical Errors	Doctor	86	74.1
	Nurse/Midwife	13	11.2
	Other Medical Staff**	8	6.9
	Medical Team	7	6.0
	Hospital Management	2	1.7
Age of Person Exposed	0 to16	45	39.8
	17 to 33	35	31.0
	34 to 50	11	9.7
	51+	22	19.5
	Total	113	100.0
Gender of Person Exposed	Female	76	66.0
	Male	40	34.0

* A large majority of neglect from the medical staff comprised of cases such as the delay of treatment, patient’s falling off a stretcher or bed, and forgetting medical equipment in the patient’s body during operation etc.

** Health Officer, Nurse, and 112 Emergency Service Medical Staff

Information in respect to the place and year that the medical errors occurred are given in Table-II. Accordingly, the maximum amount of medical errors occurred in 2013 (33.6%). While the highest number of medical errors occurred in the Marmara region with the ratio of 25.5%, and the least amount of medical errors occurred in the Black Sea region with the ratio of 6.9%. In respect to the distribution of the medical errors according to the institution, the highest ratio occurred in state hospitals with the ratio of 31.9%. In respect to the distribution of medical errors according to the units within the hospital; while surgical services were on top with the ratio of 56.0%, emergency and intensive care units ranked as the second where medical errors occurred and it accounted for 25.0% (Table-II).

**Table-II:** Findings for year and place that the medical errors occurred.

		*n*	*%*			*n*	*%*
Distribution by Year	2012	23	19.8	Distribution According to Services	Emergency and Intensive Care	29	25.0
	2013	39	33.6		Internal Services	11	9.5
	2014	28	24.1		Surgical Services	65	56.0
	2015	26	22.4		Others*	11	9.5
	Total	116	100.0		Total	116	100.0
Distribution by Region	Marmara Region	29	25.0	Distribution According to Institutions	State Hospital	37	31.9
	Marmara Region	21	18.1		Education and Research Hospital	23	19.8
	Central Anatolian Region	19	16.4		University Hospital	13	11,2
	Mediterranean Region	18	15.5		Private Hospital	32	27,6
	South-Eastern Anatolia	11	9.5		Others **	11	9.5
	Eastern Anatolian Region	10	8.6		Total	116	100.0
	Black Sea Region	8	6.9				

* Pain and Anesthesia Center, Beauty Center, Health Center, Private Polyclinic/Clinic/Medical Centre, Ambulance, non-hospital

The findings concerning how the medical errors resulted are given in Table-III. Accordingly, approximately half of the medical errors (51.7%) resulted in death, and 25.0% of them resulted in either permanent damage or injury. Only 1.7% of the medical errors that occurred resulted in tangible damage. When evaluated in respect to the people who made medical error; 52.9% of them were under investigation, and 30.4% of those were allegations (Table-III).

**Table-III:** Consequences of medical errors.

		*n*	*%*
In Terms of Patient	Death	60	51.7
	Permanent Damage/Inability State	29	25.0
	Prolongation Of Treatment	10	8.6
	Moral Damage	9	7.8
	Coma/Paralysis	6	5.2
	Tangible Damage	2	1.7
	Total	116	100.0
In Terms of People Who Made Medical Error	Investigation	54	52.9
	Made an Allegation	31	30.4
	Fine	12	11.8
	Prison Sentence	5	4.9
	Total	102	100.0

## DISCUSSION

Very few studies in the literature have investigated how medical errors are reported in the media. According to the results of this study performed with the aim of identifying the responses of the media to medical errors occurring in medical institutions in Turkey, and focusing on news concerning medical errors taking place in newspapers in the last three years; a large majority of medical errors resulted from the negligence of the medical staff. In particular, the delaying of patient’s treatments were the most common. Another important reason considered was faulty or wrong drug administration. In the study conducted by Li et al.^20^ in order to analyze the coverage of medical errors occurring in cancer patients, in the media; it was found that medication errors were one of the most common error types, and the intensity of damage exposed to a vast majority of patients were “severe”. According to the result of the research conducted by Hinchcliff et al.^15^ with the aim of analyzing the news about medication errors taking place in the media, the basic reason for medication errors was a shortage of resources.

This study revealed that the majority of people causing medical errors were physicians. The physicians were blamed as individual responsible for medical errors in 86 of 116 pieces of news taking place in the media, analyzed in the study; nurses/midwives were blamed in 13 cases. In the study performed by Li et al.^20^, the individuals most responsible for medical errors were identified as “clinicians”. In this context, it is especially important for physicians and nurses to take responsibility for patient safety and comply with the required procedures.^21^ Studies have also reported that medical errors have emotional response among the healthcare professionals involved and it also results in behaviour change among them.^22^

Another important finding from the research is that medical errors have resulted in patient death to a large extent, and permanent damage and disabilities have also occurred in patients. Medical errors are intolerable in medical services. This study also showed that while prosecutions were started against about half of the medical staff who made the error; those whose judicial process had been completed, were punished with imprisonment and fines.

While the news about medical errors in newspapers reveals information about types, causes and results of medical errors, they also reflect the media point of view. The media’s following of medical errors and reporting them as news and especially announcing them to the public could put pressure on health care institutions to report medical errors to relevant authorities to take appropriate measures. Investigation of the contents of medical error reported by the media would be important to initiate effective measures to ensure that such medical errors are minimized or their occurrence is eliminated altogether. However, it must be noted that most often it is the failure of the systems which results in medical errors, hence apart from taking action against those involved, efforts should be made to improve the healthcare delivery system and reporting of medical errors by the healthcare professionals should also be encouraged so that the administration can analyze them and then order appropriate interventions.
